# Elevated HIV risk behaviour among recently incarcerated injection drug users in a Canadian setting: a longitudinal analysis

**DOI:** 10.1186/1471-2458-9-156

**Published:** 2009-05-27

**Authors:** M-J S Milloy, Jane Buxton, Evan Wood, Kathy Li, Julio SG Montaner, Thomas Kerr

**Affiliations:** 1 British Columbia Centre for Excellence in HIV/AIDS, St. Paul's Hospital, Vancouver, Canada; 2 School of Population and Public Health, University of British Columbia, Vancouver, Canada; 3 Department of Epidemiology, British Columbia Centre for Disease Control, Vancouver, Canada; 4 Department of Medicine, University of British Columbia, Vancouver, Canada

## Abstract

**Background:**

While incarceration has consistently been associated with a higher risk of HIV infection for individuals who use injection drugs (IDU), the effect of incarceration on the post-release risk environment remains poorly described. We sought to assess the impact of incarceration on risk factors for HIV infection after release from prison in a sample of active IDU in Vancouver, Canada.

**Methods:**

Using a prospective cohort of community-recruited IDU followed from May 1, 1996 to November 30, 2005, we examined contingency tables and performed linear growth curve analyses to assess changes in the prevalence of independent risk factors for HIV infection from before to after a period of incarceration among participants reporting incarceration and a matched control group.

**Results:**

Of the 1603 participants followed-up over the study period, 147 (9.2%) were eligible for an analysis of post-incarceration risk behaviours and 742 (46.3%) were used as matched controls. Significant differences were found in one or both groups for the prevalence of frequent cocaine injection, requiring help injecting, binge drug use, residence in the HIV outbreak epicentre, sex-trade participation and syringe sharing (all *p *< 0.05) after incarceration. In linear growth curve adjusted for age, gender and ethnicity, syringe sharing was significantly more common in those recently released from prison (*p *= 0.03) than in the control group.

**Conclusion:**

In a sample of Canadian IDU, we did not observe any effect of incarceration on the prevalence of several behaviours that are risk factors for HIV infection, including intensity of drug use or participation in the sex trade. However, those recently released from prison were more likely to report syringe sharing that those in a matched control group.

## Background

Incarceration is common among injection drug users (IDU) and has consistently been associated with drug-related harms, especially infection with blood-borne pathogens like hepatitis C and HIV. [1-3] While many IDU cease drug use upon imprisonment,[4,5] those that persist do so in environments of elevated risk. In many penal facilities, including those in the United States, Canada, Australia and the United Kingdom, harm reduction measures, such as the distribution of sterile syringes, are unavailable and the possession of injection equipment is outlawed. [6,7] Epidemiological surveys of prisoners in a variety of settings, including the United Kingdom, [8-11] Greece[12] and Thailand[13,14] identified endemic use of contaminated contraband syringes. Contact tracing investigations found this dynamic fuelled prison-based HIV outbreaks in Australia,[15] Russia,[16] Lithuania[17] and Scotland. [1]

Although the link between imprisonment and HIV infection is robust and well described, the possible effects of incarceration on post-release behaviours of IDU and their HIV risk environment remain largely undetermined. [18] Findings from related inquiries into sexual risk factors for ex-prisoners suggest the experience of incarceration, transition to non-correctional settings and reintegration into communities all influence post-release behaviours. [19-21] While these studies are primarily concerned with individual-level sexual risks, newly-released prisoners often face difficulties finding employment,[22,23] securing housing,[22,24] reestablishing social supports,[25,26] accessing healthcare,[22,27] and enduring discrimination,[28] and these factors are known to structure HIV risk. [29,30] However, very little attention has been paid to the post-release trajectory of IDU or the specific determinants of their risk environment.

We are unaware of any analyses that identify the effect of incarceration on the risk factors for HIV infection following release from prison or analyses that describe the specific individual, social and structural determinants of the post-release risk environment for IDU. Thus, in the current analysis, we sought to determine the possible effect of imprisonment on the post-release risk environment by identifying the prevalence of independent risk factors for HIV infection before and after a period of incarceration as compared to a non-incarcerated matched control group.

## Methods

In May 1996, the Vancouver Injection Drug User Study (VIDUS) began recruiting IDU through self-referral and street outreach. This prospective cohort study has been previously been described in detail. [31] In brief, individuals were eligible for recruitment if they had injected drugs at least once in the previous month, resided in greater Vancouver and provided written informed consent. At baseline and every six months, participants provide venous blood samples and complete an interviewer-administered questionnaire. This structured questionnaire elicits demographic data, information about recent drug use patterns, HIV risk behaviours, encounters with the criminal justice system and experiences in addiction treatment and other health-care settings. All participants are given a $20 stipend at each visit. This study has received approval from the Providence Health Care/University of British Columbia Research Ethics Board.

To inform our analysis of HIV risk factors, we first identified all participants who reported being incarcerated in a municipal jail, provincial prison or federal penitentiary overnight or longer since initiating injection drug use, a definition consistent with previous analyses. [32,33] Among these individuals, only those who had completed a study visit both before and after this incarceration episode were eligible for an analysis of post-release risk behaviour. Using frequency matching, participants who had completed identical follow-up visits and did not report recent incarceration at all during this period were included in the control group. (Controls could have been incarcerated at other times during the follow-up period.)

We then examined if there were significant differences between the two groups with regards to age, gender and ethnicity (Aboriginal vs. non-Aboriginal) using χ^2 ^tests and Wilcoxon rank-sum tests. We repeated these tests to compare individuals included as cases or controls to participants not included in the final analytic sample.

Next, we selected explanatory variables that had previously been identified as implicated in the transmission of HIV in this setting: daily cocaine injection (yes vs. no);[34] daily injection of a mixture of heroin and cocaine ("speedball") (yes vs. no);[35] requiring help injecting (yes vs. no);[36,37] binge drug use (yes vs. no);[38] unstable housing (yes vs. no);[34] having sought and been denied addiction treatment (yes vs. no);[39] residence in the city's HIV epicenter, the Downtown Eastside (DTES) (yes vs. no);[31] public drug use (yes vs. no);[40] participation in the sex trade (yes vs. no);[36] and syringe sharing (yes vs. no). We also included consistent condom use with regular sexual partners (yes vs. no) and consistent condom use with casual sexual partners (yes vs. no). As in previous work, frequent drug use was defined as once or more per day. [41] Unstable housing was defined as living in a single-room occupancy hotel room, a shelter, or being homeless. [41] All variables referred to the previous six months except unstable housing, which referred to current conditions.

To test for differences between pre- and post-incarceration periods between those incarcerated and the control group, we used McNemar's test to examine the proportion of individuals reporting each risk factor in each group. To test for differences over time and between groups, we constructed linear growth curve models for each risk factor in which a statistically significant trend was observed in one or both groups. Commonly used in longitudinal observational research, the linear growth curve technique enables the identification of changes over time by using an interaction term in the model to determine if those changes are significant. [42,43] In each linear growth curve model, the slope represents the differences in outcomes by group (incarcerated vs. control) over time (before vs. after); the *p*-value represents the significance of the interaction term. In addition, to control for potential confounding, an *a priori *model fitting approach was used in which each model was adjusted for age, gender and ethnicity (Aboriginal vs. non-Aboriginal). All *p*-values were two-sided.

## Results

**Figure 1:**
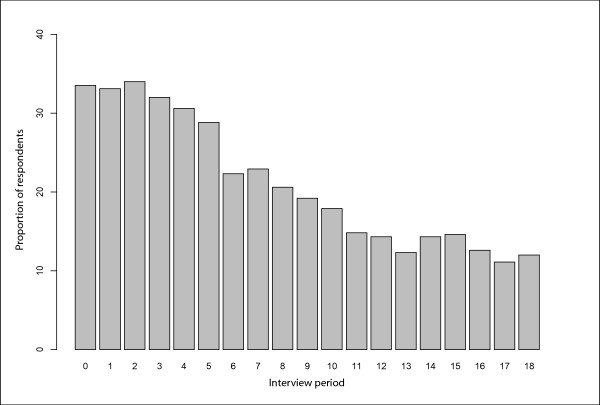
**"Prevalence of individuals reporting incarceration in the previous six months at baseline and each follow-up period in VIDUS"**.

Between May 1, 1996 and November 30, 2005, 1,603 participants were recruited, including 584 (36.4%) women and 435 (27.1%) people who reported Aboriginal ancestry. At baseline, the median age of the participants was 33.0 (IQR = 26.0–40.0). The proportion of respondents at baseline and each follow-up period who reported incarceration in the previous six months is presented in Fig 1. Among the study participants, 147 (9.2%) individuals reported an incarceration event and completed a follow-up survey both before and after the event. Of the remainder, 742 (46.3%) did not report an incarceration event at identical follow-up periods; they were included in the matched control group. Participants in the matched control group had a median age of 35.5 (IQR: 28.8 – 41.3), significantly older than the incarceration group (median age: 33.4, IQR: 26.3 – 39.0, *p*-value < 0.01). The groups did not differ significantly with respect to gender or ethnicity. In tests comparing individuals included as cases or controls to cohort participants not included in the analyses, we found those individuals not included were significantly younger (median age at baseline of 30.0, IQR = 23.9 – 37.7 compared to median age at baseline of 35.1, IQR = 28.0 – 41.1, *p *< 0.001) and more likely to be female (39.2% compared to 32.9%, *p *= 0.01). The observed difference in the proportions of individuals reporting aboriginal ancestry (24.6% among non-included compared to 29.1%) was not statistically significant (*p *= 0.051).

The proportion of each group reporting HIV risk factors in each period, as well as the result of McNemar's test assessing whether the proportions within groups were equal, is reported in Table 1. No significant differences between the before and after period were observed for either the incarcerated or control group in the prevalence of frequent speedball injection, living in unstable housing, being denied addiction treatment, public drug use or consistent condom use with regular or casual sexual partners (all *p *> 0.05). Thus, these factors were not included in the linear growth curve analyses. For frequent cocaine injection, a significant decrease was observed in the control group (*p *< 0.001); the decrease among the incarcerated group was not statistically significant. A statistically significant decrease in needing help injecting was observed in both the incarcerated and control group (both *p *< 0.001). For binge drug use, a statistically significant decrease was observed in both the incarcerated (*p *< 0.005) and control (*p *< 0.001) groups. Fewer individuals in both the incarcerated (*p *= 0.016) and control (*p *= 0.011) groups reported living in the DTES. A significantly lower proportion of individuals reported participating in the sex trade in the incarcerated (*p *= 0.012) and matched (*p *< 0.001) groups. A statistically significant decrease in the prevalence of syringe sharing was observed in the non-incarcerated group (*p *< 0.001); the decrease in the incarcerated group was not statistically significant (*p *= 0.398).

**Table 1:** Risk factors for HIV infection among incarcerated cases (*n *= 147) before and after a period of incarceration compared to matched controls (*n *= 742)

Risk factor	Before		After		*p*-value*
		
	*n*	%	*n*	%	
**Frequent cocaine injection**					
Incarcerated	46	31.3	37	25.2	0.170
Matched control	205	27.6	150	20.2	**< 0.001**
**Frequent speedball injection**					
Incarcerated group	12	8.2	17	11.6	0.297
Matched control	70	9.4	67	9.0	0.748
**Need help injecting**					
Incarcerated group	46	31.3	27	18.4	**0.001**
Matched control	202	27.2	142	19.1	**< 0.001**
**Binge drug use**					
Incarcerated group	66	44.9	45	30.6	**0.005**
Matched control	271	36.5	210	28.3	**< 0.001**
**Unstable housing**					
Incarcerated group	100	68.0	101	68.7	0.889
Matched control	418	56.3	414	55.8	0.806
**Denied addiction treatment**					
Incarcerated group	24	16.3	19	12.9	0.411
Matched control	95	12.8	81	10.9	0.230
**Resident in the DTES**					
Incarcerated group	96	65.3	78	53.1	**0.016**
Matched control	377	50.8	343	46.2	**0.011**
**Public drug use**					
Incarcerated group	32	21.8	22	15.0	0.114
Matched control	116	15.6	110	14.8	0.602
**Sex trade participation**					
Incarcerated group	36	24.5	25	17.0	**0.012**
Matched control	154	20.8	116	15.6	**< 0.001**
**Syringe sharing**					
Incarcerated	38	25.9	33	22.5	0.398
Matched control	231	31.3	133	17.9	**< 0.001**
**Condoms w/casual partners**					
Incarcerated	27	18.4	23	15.7	0.505
Matched control	116	15.6	103	13.9	0.312
**Condoms w/regular partners**					
Incarcerated	30	20.4	28	19.1	0.739
Matched control	133	17.9	113	15.2	0.121

* *p*-value associated with McNemar's test of equality

The results of the linear growth curve analyses are presented in Table 2. In models adjusted for age, gender and Aboriginal ethnicity, no significant differences were found in the prevalence of frequent cocaine injection (*p *= 0.737); needing help injecting (*p *= 0.201); binge drug use (*p *= 0.273); living in the DTES (*p *= 0.105) or participation in the sex trade (*p *= 0.623) before and after a period of incarceration compared to a matched control group. However, in the linear growth curve analysis of syringe sharing, a significant difference (*p *= 0.033) was observed between the slopes for the incarcerated and control groups, indicating a significant decrease in prevalence in the control group but no significant decrease in the incarcerated group.

**Table 2:** Linear growth curve analyses of HIV risk factors modelled as outcome, adjusted for age, gender and ethnicity (Aboriginal vs. non-Aboriginal)

Risk factor	Slope (95% Confidence Interval)	*p*-value
**Frequent cocaine injection**		
Incarcerated	-0.342 (-0.792, 0.107)	0.737
Matched control	-0.403 (-0.595, -0.212)	
**Need help injecting**		
Incarcerated	-0.777 (-1.218, -0.335)	0.201
Matched control	-0.467 (-0.665, -0.269)	
**Binge drug use**		
Incarcerated	-0.630 (-1.047, -0.212)	0.273
Matched control	-0.373 (-0.584, -0.162)	
**Resident in the DTES**		
Incarcerated	-0.574 (-1.007, -0.142)	0.105
Matched control	-0.185 (-0.328, -0.042)	
**Sex trade participation**		
Incarcerated	-0.575 (-1.041, -0.109)	0.623
Matched control	-0.398 (-0.609, -0.186)	
**Syringe sharing**		
Incarcerated	-0.217 (-0.657, 0.224)	**0.033**
Matched control	-0.722 (-0.933, -0.512)	

## Discussion

In this analysis, we found individual, social and structural risk factors for HIV infection were common among active IDU both before and after periods of incarceration. In a linear growth curve analysis, individuals recently released from prison were significantly more likely to report sharing contaminated sharing syringes as compared to individuals who did not report incarceration.

This finding sheds further light on the relationship between incarceration and the ongoing HIV epidemic among IDU in this setting. Both qualitative[44] and quantitative [32-34] findings from two prospective cohorts of IDU in Vancouver have confirmed the link between incarceration and an elevated risk of infection. [31,34] In a longitudinal analysis of incident cases, individuals reporting incarceration were more than twice as likely to become infected with HIV;[34] incarceration was also independently associated with syringe sharing. [32,33] As a result of these risks and the high prevalence of incarceration among local IDU,[32] 21% of HIV cases in the Vancouver outbreak are estimated to be the result of imprisonment. [45]

Although we were unable to determine the exact nature of the relationship, these findings suggest that some aspect of the environment or experience of incarceration leads to a greater risk of syringe sharing once individuals are released from custody. Many previous analyses from this setting and others have identified a greater risk of syringe sharing among imprisoned individuals compared with analagous samples of IDU, especially in correctional environments where access to sterile syringes is forbidden. [10,32] In the current analysis, perhaps individuals exposed to correctional environments are normalized to the practice while incarcerated and continue once released. [44] It is also possible that the uncertainty and instability that characterizes the immediate post-release period interrupts access to or use of harm reduction services. Among non-incarcerated individuals, the decreased level of syringe sharing observed might be related to the expansion of opioid substitution therapies and other harm reduction services in this setting, including the opening of a supervised injection facility. [46] Given the observational nature of our analyses, our current findings are exploratory and should spur further research to evaluate these hypotheses pertaining to post-release sharing.

To our knowledge, this is the first analysis to evaluate the effect of incarceration on post-release rates of syringe sharing. Two previous studies measured the prevalence of syringe sharing following release from prison in Bangkok, Thailand[2] and New South Wales, Australia. [47] In Thailand, HIV-positive cases were significantly more likely (AOR = 2.9, 95% Confidence Interval: 1.7 – 5.0) to report borrowing syringes in the month following incarceration compared to HIV-negative controls. In Australia, a larger proportion of HIV-positive cases (20%) than HIV-negative controls (15%) reported syringe sharing after discharge from prison. However, in neither study could the effect of incarceration be determined as a non-incarcerated control group was not included; the comparison group was constructed by serostatus. In our study, the use of a non-incarcerated control group allowed the identification of the independent effect of imprisonment.

We did not observe an effect of imprisonment on any of the risk factors also classified as crimes in our setting, including participation in the sex trade and illicit drug use. This is in line with previous analyses that found many IDU resume drug use following release[48] and that there is no empirical evidence to support the use of enforcement to reduce the population prevalence of drug use. [49]

These findings suggest a need for a number of policy reforms. As urged by other authors, we suggest that political and criminal justice interventions that seek to reduce drug use through law enforcement should be considered in light of our findings and others linking imprisonment and a greater likelihood of HIV risk behaviours. [4,32,33,44] On a practical level, federal and provincial prison authorities should expand the in-prison availability of methadone maintenance therapy, recently shown in a randomised control trial to improve treatment and drug-use outcomes for recently-released prisoners,[50] and promote post-release support and referral to harm reduction opportunities, including needle exchange, addictions treatment and the city's supervised injection facility.

This study has methodological limitations to consider when evaluating the findings. First, VIDUS is not a random sample, although it is believed to be representative of the local population of IDU. Second, although several of the surveyed behaviours may be under- or over-reported due to social desirability, we do not believe they were differentially reported by incarceration history. Although information on HIV serostatus is available for all participants, the number of incident infections during the study period among individuals included as cases or controls is too low to allow for a valid statistical analysis; thus, we have used measures of HIV risk to evalute the possible effects of incarceration on post-release transmission patterns. Most importantly, there may be differences between recently incarcerated and non-incarcerated IDU that we were unable to account for in multivariate analyses. Finally, we were not able to include the length or location (i.e., municipal, provincial or federal) of incarceration events, nor the number of incarceration events lifetime or during the study period, in these analyses.

## Conclusion

To conclude, we evaluated the prevalence of independent risk factors for HIV infection before and after incarceration among active IDU, and, after comparison with a matched control group, observed a statistically significant relationship between syringe sharing and the post-release period. We did not find any association between incarceration and the frequency of other individual, social and structural factors, including those also defined as crimes. These findings point to the need for the ongoing development of programs both within and following prison that aim to reduce risk behaviour among IDU exposed to correctional environments.

## Competing interests

M-J Milloy, Jane Buxton, Evan Wood, Kathy Li and Thomas Kerr declare they have no competing interests. Julio Montaner has received educational grants from, served as an *ad hoc *adviser to or spoken at various events sponsored by Abbott Laboratories, Agouron Pharmaceuticals Inc., Boehringer Ingelheim Pharmaceuticals Inc., Borean Pharma AS, Bristol-Myers Squibb, DuPont Pharma, Gilead Sciences, GlaxoSmithKline, Hoffmann-La Roche, Immune Response Corporation, Incyte, Janssen-Ortho Inc., Kucera Pharmaceutical Company, Merck Frosst Laboratories, Pfizer Canada Inc., Sanofi Pasteur, Shire Biochem Inc., Tibotec Pharmaceuticals Ltd. and Trimeris Inc.

## Authors' contributions

TK and M-JM conceived the study. EW, TK, M-JM and KL designed the analysis; KL performed the statistical procedures. M-JM wrote the manuscript and incorporated all suggestions. JM contributed to conception and design of the analysis, interpretation of the data and drafting of the report. All authors approved the version to be published.

## Pre-publication history

The pre-publication history for this paper can be accessed here:


